# Sulcus-Seated Modified Tofflemire Matrix Enables the Crown Lengthening-Free Restoration of a Deep Cervical Lesion on a Maxillary Canine: A Case Report

**DOI:** 10.7759/cureus.93317

**Published:** 2025-09-26

**Authors:** Fahad A Malik, Arthur R Volker, Aiman Shahbaz

**Affiliations:** 1 Dentistry, Lahore Medical and Dental College, Lahore, PAK; 2 General Dentistry, Dent-Care Dental, New York, USA; 3 Dentistry, Columbia University, New York, USA

**Keywords:** cervical lesion, deep margin elevation, resin composite, subgingival restoration, tofflemire matrix

## Abstract

Deep cervical lesions with subgingival margins are traditionally managed by crown lengthening; however, this surgery can compromise aesthetics and the periodontium. This report describes a minimally invasive technique that combines a custom-trimmed Tofflemire matrix with modern adhesive and thermally enhanced composite placement to relocate the margin coronally and restore the defect without surgery. A 63-year-old healthy woman presented with a non-carious cervical lesion on the maxillary left canine (FDI #23). After infiltration with 1.8 mL of 3% mepivacaine (without epinephrine), a stainless-steel Tofflemire band was shortened to ~2 mm height and seated interproximally until gingival blanching confirmed sulcular sealing. The enamel-dentin interface was etched with 35% phosphoric acid, rinsed, and primed with two coats of a fifth-generation adhesive (Prime & Bond NT (Dentsply Sirona, Charlotte, North Carolina, United States)) followed by light-curing. A thin layer of flowable composite "wetted" the surface, and then a preheated nanohybrid composite (Filtek Supreme Ultra, Body A2, 68°C (3M, Saint Paul, Minnesota, United States)) was injection-molded into the matrix and incrementally light-cured from multiple angles. After matrix removal, finishing and polishing were performed with fine diamond burs and multi-step rubber polishers. The modified matrix provided stable isolation, enabling precise adhesive placement and warmed composite buildup in a single visit. Clinical inspection revealed a flush cervical margin, healthy gingiva, and an anatomically accurate emergence profile. The patient reported no postoperative discomfort, and no surgical intervention was required. A simple chairside modification of the Tofflemire matrix can facilitate predictable sulcular isolation and margin elevation in subgingival cervical lesions, offering a conservative, single-visit alternative to surgery. Further clinical studies with larger samples and long-term follow-up are needed to validate this approach.

## Introduction

Root surface caries and cervical lesions are increasingly common as populations age [1,2]. Restoring a lesion that extends below the gingival margin presents significant challenges in moisture control, matrix adaptation, and periodontal considerations. Without a supragingival margin, traditional matrix placement may be distorted by wedging, leading to open margins or overhangs [3-5]. Additionally, subgingival carious defects risk violating the biologic width, the physiologic space occupied by the junctional epithelium and connective tissue attachment, which can complicate achieving a proper emergence profile. The standard approach for deep cervical or proximal lesions often involves surgical crown lengthening to expose sound structure. However, surgery can be invasive, costly, and undesirable in aesthetic areas or when adjacent dental implants are present.

Advances in adhesive dentistry have introduced the concept of cervical margin relocation or deep margin elevation, a minimally invasive technique where the cavity margin is elevated coronally with a restorative base to create supragingival conditions for adhesive restoration [6,7]. This method allows clinicians to restore subgingival cavities in a controlled field, serving as a minimally invasive alternative to surgical crown lengthening [6]. A key step in deep margin elevation is achieving isolation and adaptation of a matrix at the deep margin. One described approach is the use of a modified Tofflemire (universal) matrix band that is trimmed and contoured to extend deep into the sulcus, effectively sealing out crevicular fluid and blood. Brackett et al. demonstrated that such a modified matrix technique can isolate subgingival root caries sites for restoration with resin-modified glass ionomer (RMGI) or composite, avoiding contamination and providing a sound foundation for adhesive buildup [3]. Similarly, earlier reports by Chan and Adkins and others outlined techniques using matrix bands in conjunction with retraction cords or glass ionomer "open-sandwich" bases to manage subgingival cervical lesions [4,5].

This case report follows the Case Report (CARE) guidelines and details the management of a deep cervical carious lesion on an upper canine (FDI #23) using a modified Tofflemire matrix. The objective is to illustrate step-by-step how this technique preserves tooth structure and periodontal health while achieving a durable restoration. We also discuss material selection (resin composite vs. glass ionomer) and the clinical outcomes in light of current evidence. By sharing this case, we aim to advance the clinical understanding of matrix modification techniques for subgingival restorations, especially in the aesthetic zone where conservative management is paramount.

## Case presentation

A 63-year-old woman presented with a concern about the appearance of her anterior teeth. She was in good overall systemic health and reported daily use of a beta-blocker and multivitamins. The patient did not report hypersensitivity or pain in the area but was dissatisfied with the aesthetic appearance of her maxillary left canine (FDI #23). Clinical examination revealed a well-demarcated, non-carious cervical lesion (NCCL) located on the facial aspect of tooth FDI #23. The lesion measured approximately 2 mm incisogingivally, was wedge-shaped, and extended slightly subgingivally, with no signs of active caries, pulpal involvement, or inflammation of the adjacent gingival tissues. Periodontal probing depths were 3-4 mm circumferentially, and the estimated distance from the gingival margin to the alveolar crest was ~5 mm. Clinical testing revealed no sensitivity to thermal (cold or heat) or mechanical (percussion and probing) stimuli. The diagnosis was consistent with a multifactorial etiology, including toothbrush abrasion and occlusal stress-induced abfraction.

After obtaining informed consent, local anesthesia was achieved with 1.8 mL of 3% mepivacaine (without epinephrine) via local infiltration adjacent to tooth FDI #23. Adequate anesthesia was confirmed after approximately two minutes before proceeding. It was then decided to restore the lesion using a conservative, non-invasive technique involving a modified Tofflemire matrix and injection-molded, heated resin composite. The enamel-dentin interface was etched with 35% phosphoric acid, rinsed, and primed with two coats of a fifth-generation adhesive (Prime & Bond NT (Dentsply Sirona, Charlotte, North Carolina, United States)) following the manufacturer's instructions and light-cured. The composite was adapted with an Ivolsult instrument (Ivoclar Vivadent, Schaan, Liechtenstein) to ensure proper marginal adaptation. The nanohybrid composite resin (Filtek Supreme Ultra (3M, Saint Paul, Minnesota, United States)) was preheated to approximately 68°C for three minutes using a composite warming device (Calset, AdDent Inc., Danbury, Connecticut, United States) to enhance flowability and adaptation. The material was incrementally placed and polymerized using an LED curing unit (Bluephase® N, Ivoclar Vivadent, Schaan, Liechtenstein) at 1200 mW/cm² for 20 seconds per increment.

**Figure 1:**
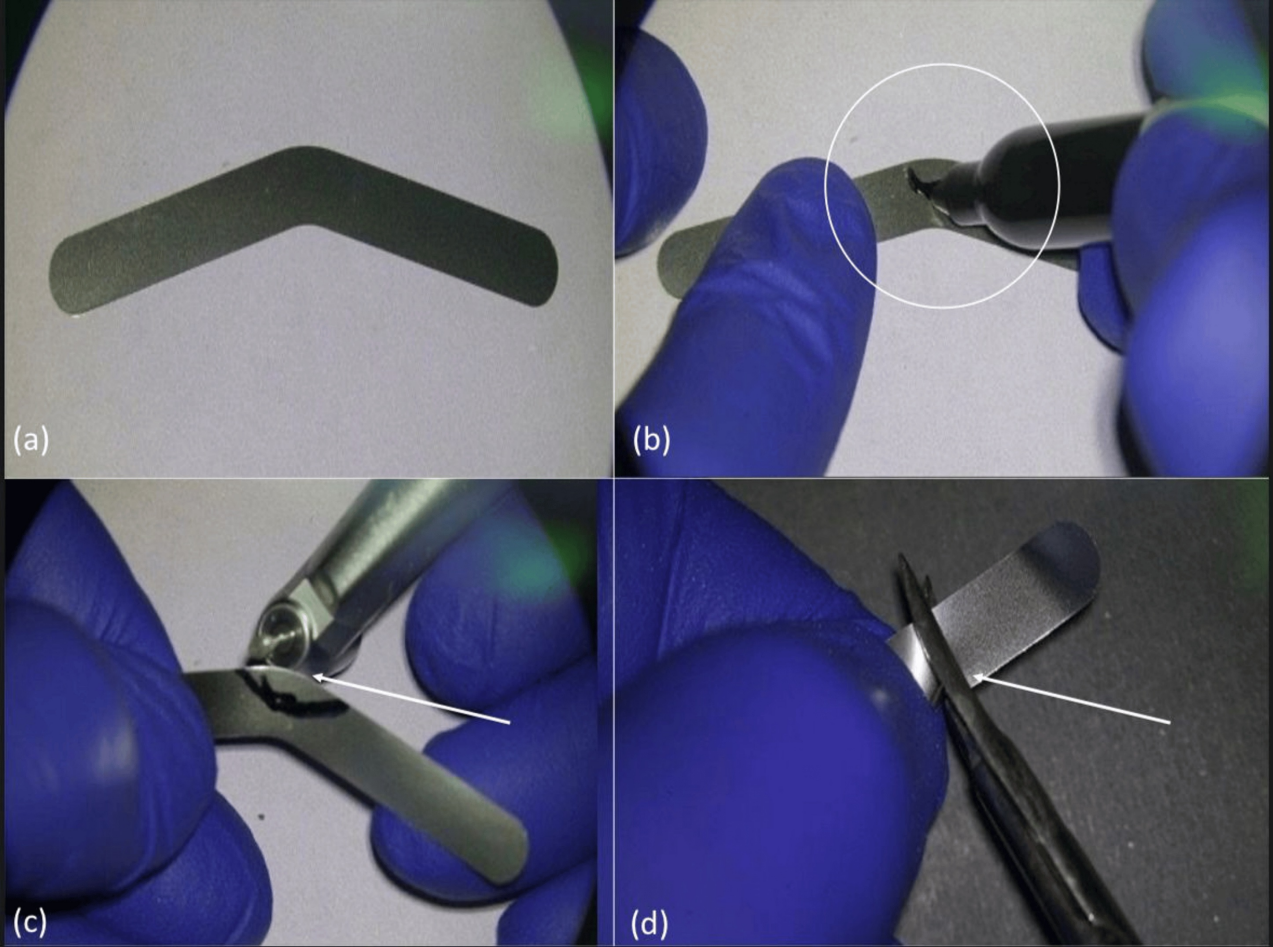
Stepwise preparation of the modified Tofflemire matrix (a) Standard matrix. (b) Curvature marked. (c) Trimming with a bur. (d) Excess removed.

A stainless-steel Tofflemire band was selected and customized for the procedure (Figure 1a). A marker was used to outline the curvature before trimming (Figure 1b). The matrix was shaped using a coarse diamond bur (Figure 1c), and excess material was removed with scissors (Figure 1d).

**Figure 2:**
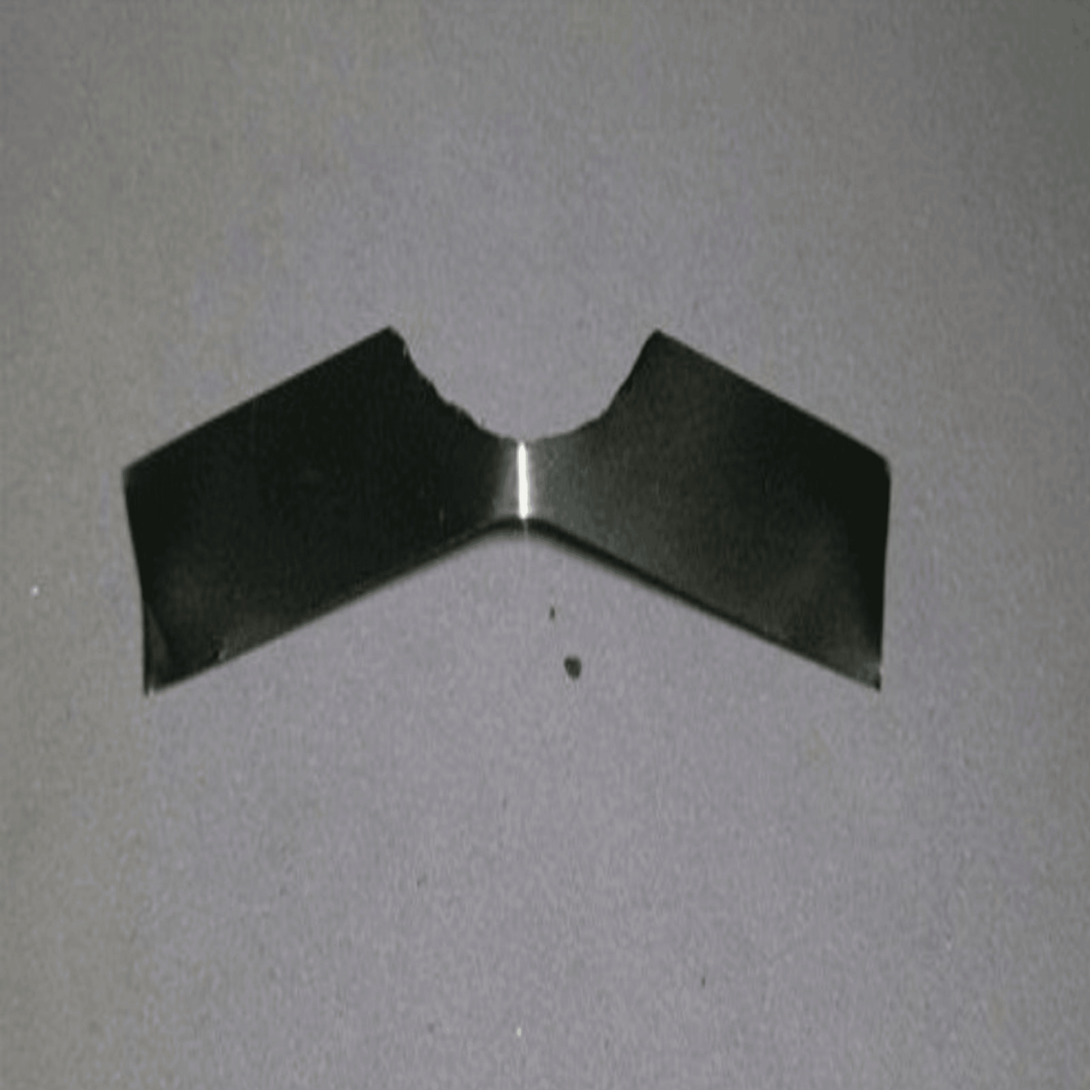
Trimmed matrix Final modified Tofflemire matrix.

Figure 2 demonstrates the final shape of the modified Tofflemire matrix.

**Figure 3:**
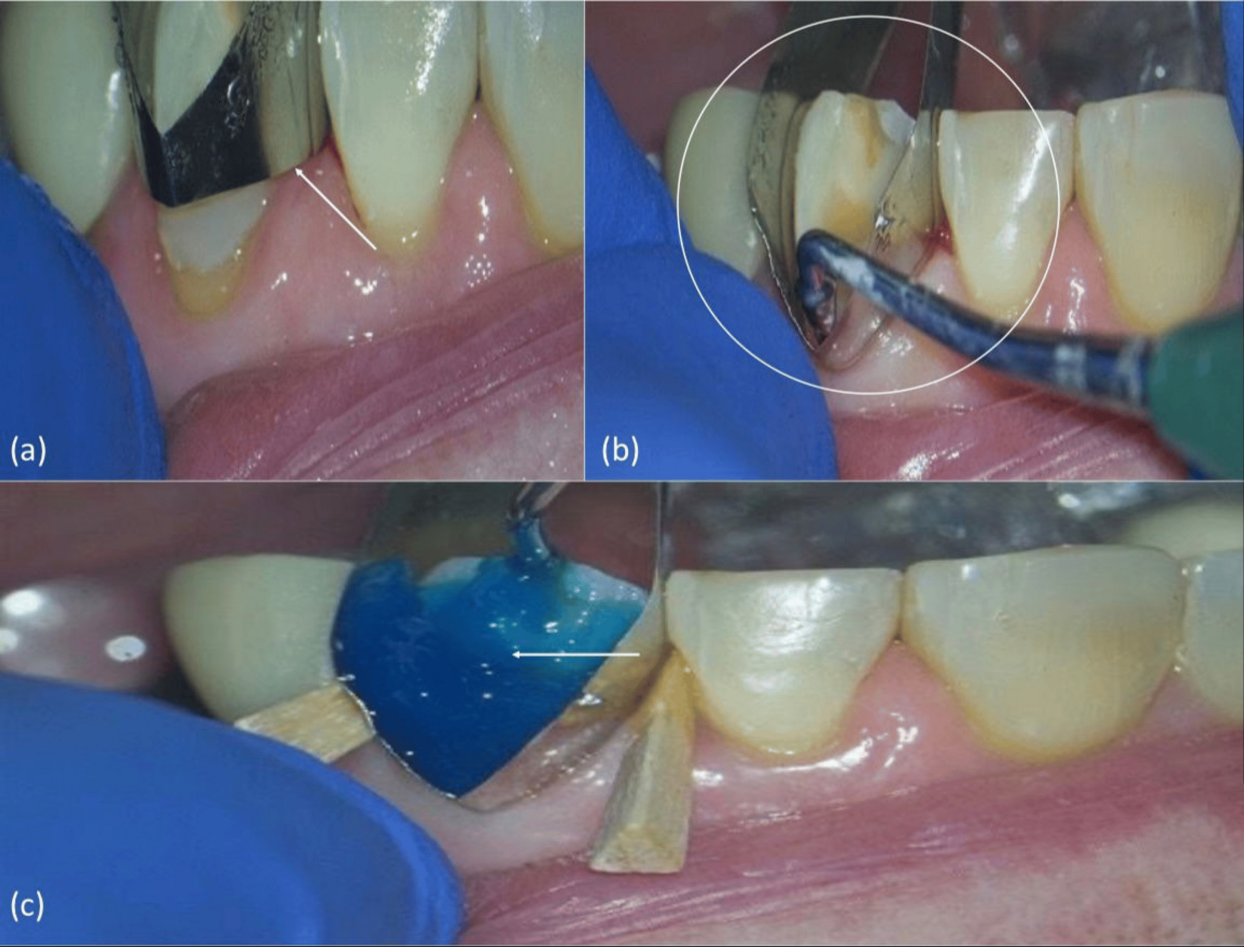
Placement and seating of the modified Tofflemire matrix (a) Insertion of the customized band. (b) Seating confirming the sulcular seal. (c) Application of the 35% phosphoric acid etchant.

The modified Tofflemire matrix was placed interproximally and gently tightened around the tooth to achieve soft-tissue displacement and firm subgingival adaptation (Figure 3a). Tissue blanching confirmed proper adaptation and sulcular sealing (Figure 3b). With the matrix stabilized, the exposed cervical surface was etched with 35% phosphoric acid gel for 15 seconds (Figure 3c).

**Figure 4:**
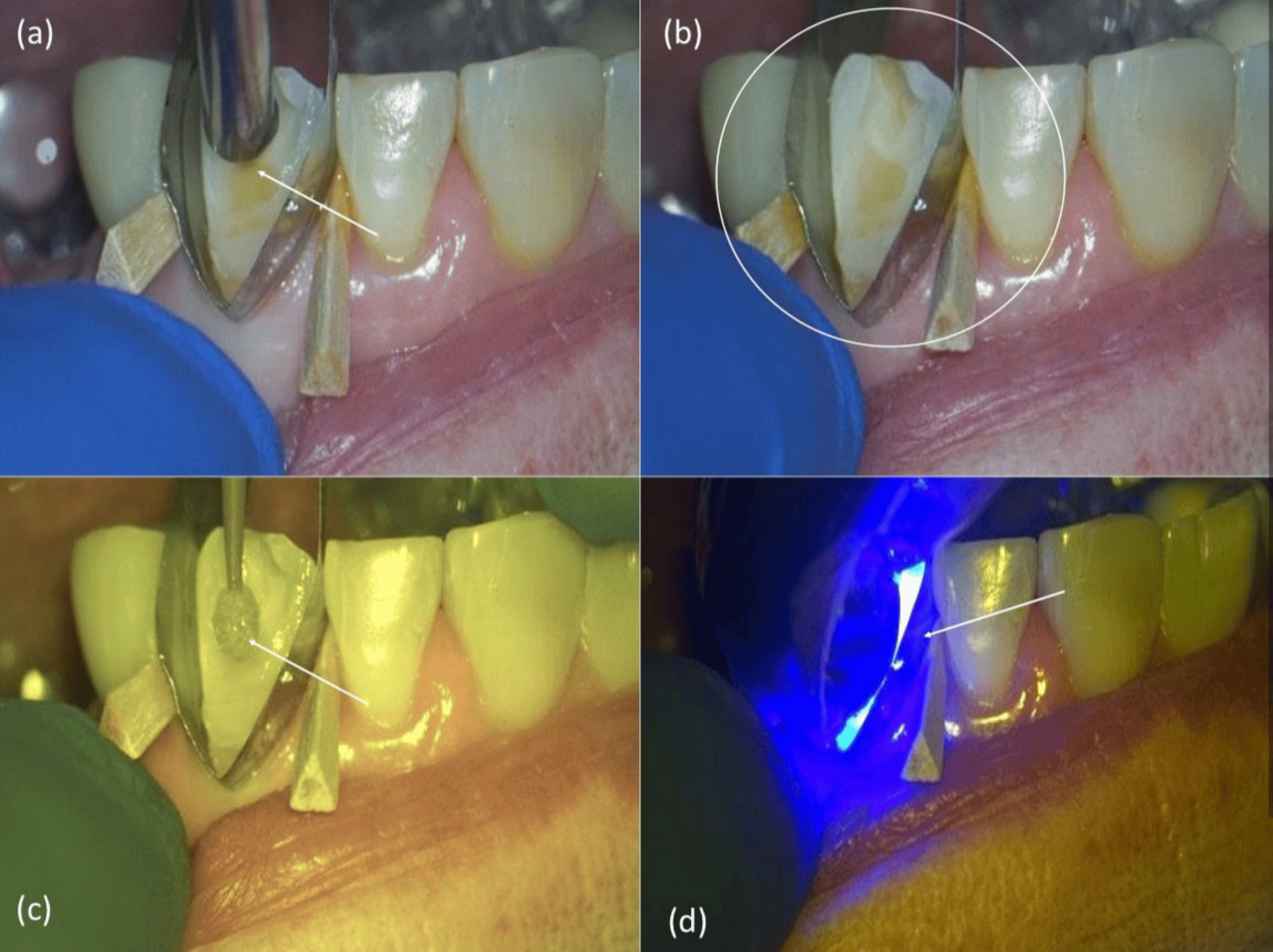
Stepwise adhesive procedure (a) Rinsing after etching. (b) Post-rinse enamel surface. (c) Application of the bonding agent. (d) Light-curing of the adhesive.

After etching, the surface was thoroughly rinsed (Figure 4a). Post-rinse evaluation showed a frosty enamel surface indicating successful etching and adequate isolation (Figure 4b). A bonding agent was then applied uniformly to the conditioned enamel and dentin (Figure 4c) and light-cured with an LED unit (Figure 4d).

**Figure 5:**
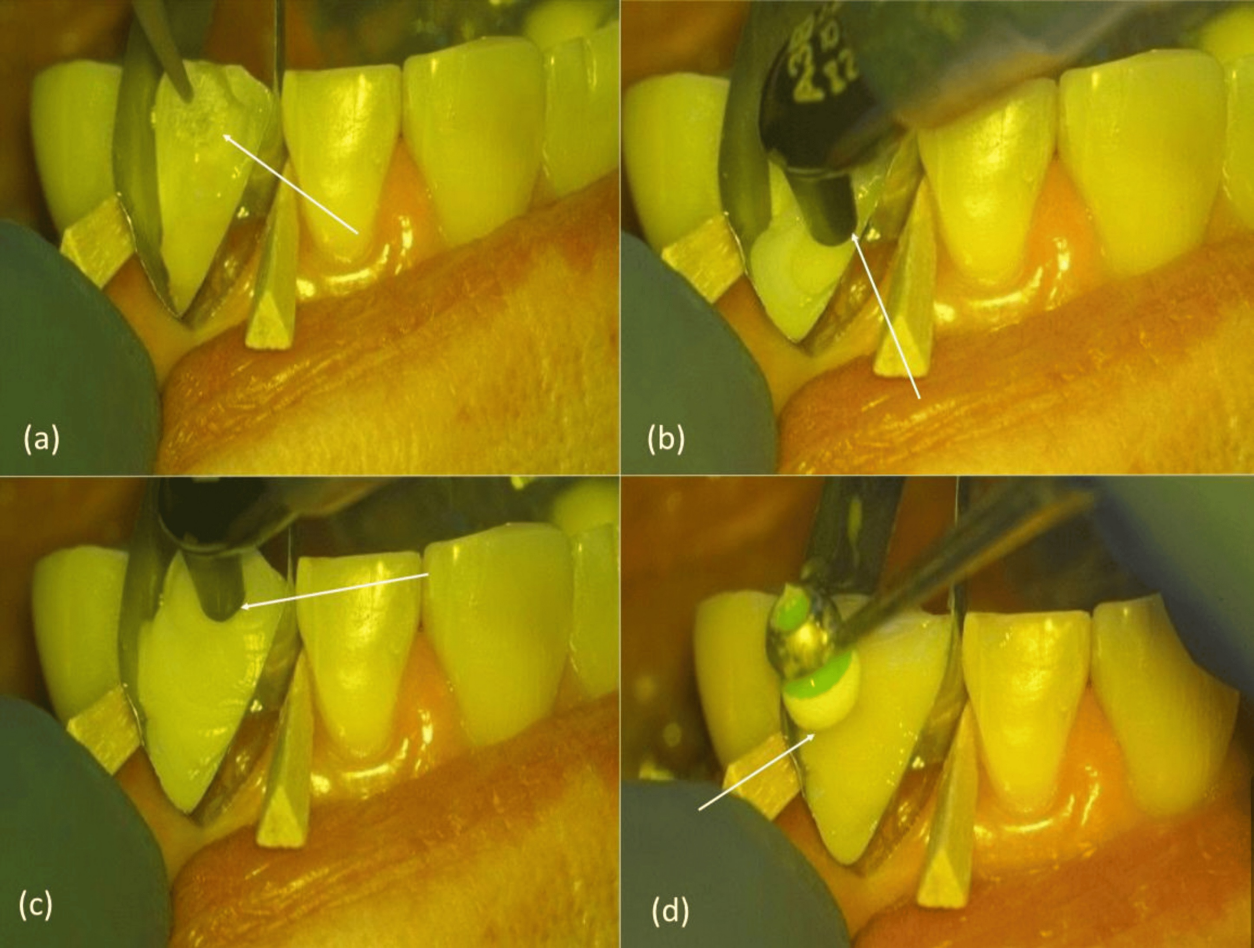
Composite placement and adaptation (a) Application of the flowable composite. (b) Injection of the preheated composite: first increment. (c) Injection of the preheated composite: second increment. (d) Final adaptation and contouring of the heated composite.

After the polymerization of the bonding agent, a small amount of flowable composite resin was applied with a microbrush to "wet" the interface and improve resin adaptation (Figure 5a). The preheated nanohybrid composite (Filtek Supreme Ultra Body, A2 shade) was then injected into the matrix in controlled increments using the injection-molding technique (Figure 5b-5c), which allowed the resin to flow evenly into the sulcus and against the matrix, minimizing voids and internal stress. Finally, the adaptation phase was performed using an Ivolsult instrument to contour the heated composite into the prepared subgingival Class V lesion (Figure 5d), ensuring intimate adaptation against cavity walls, improving marginal seal, and maintaining ideal sculptability.

**Figure 6:**
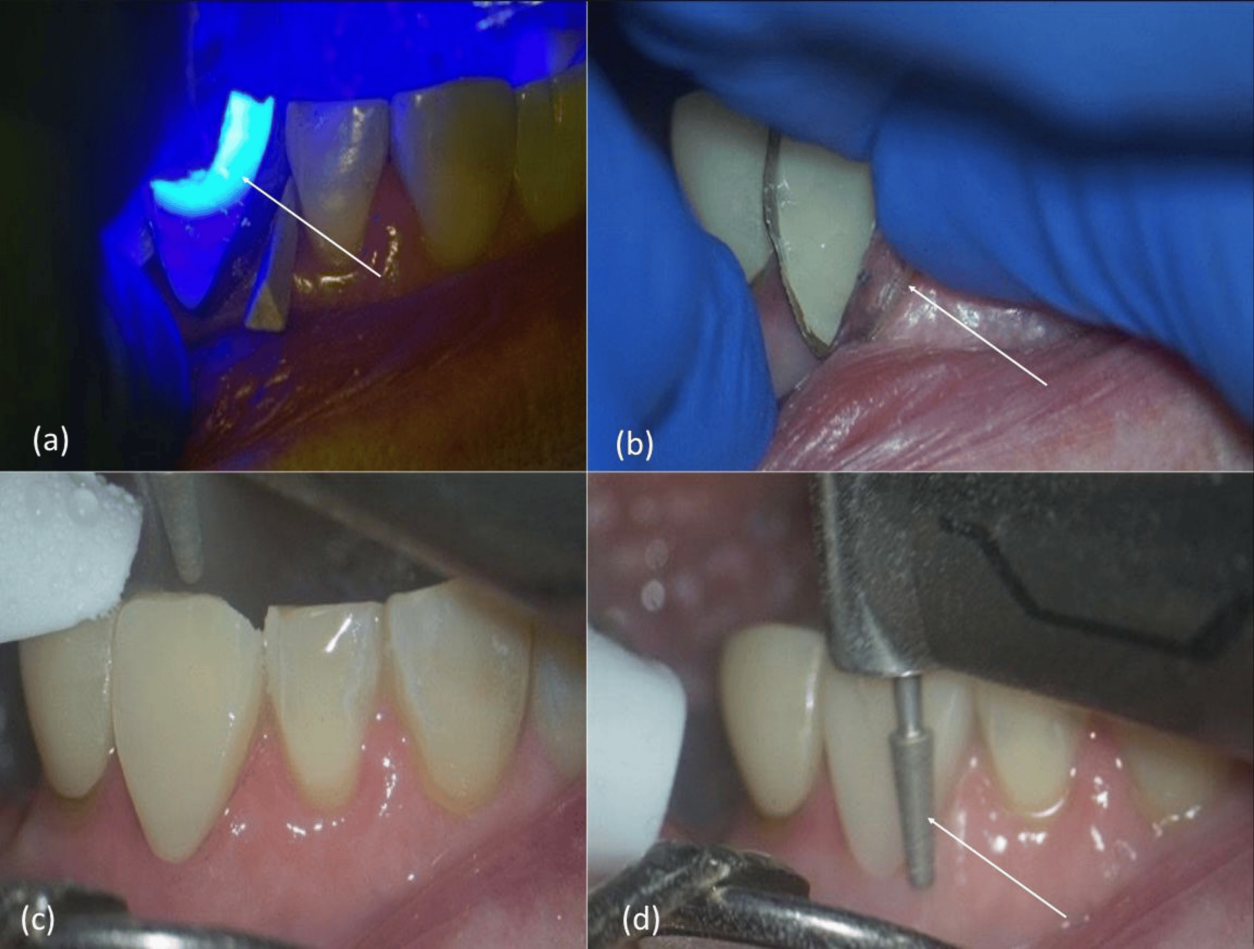
Final light-curing, matrix removal, finishing, and polishing (a) Final light-curing using LED. (b) Removal of the customized matrix band. (c) Finishing the restoration. (d) Polishing with a fine-tapered bur.

Incremental light-curing was performed from multiple angles (facial, incisal, and lingual) to ensure complete polymerization (Figure 6a). After curing, the customized Tofflemire matrix band was removed carefully without disrupting the newly formed margins, revealing the final adaptation of the restoration and confirming successful reproduction of the original tooth morphology and emergence profile; additional light-curing was performed under a glycerin-based gel to eliminate the oxygen-inhibited layer (Figure 6b). Finishing and contouring were then completed using a flame-shaped diamond bur to reproduce natural anatomic form and achieve smooth transitions with adjacent tooth surfaces (Figure 6c). Polishing was carried out with a multi-step rubber polishing system using medium and fine FlexiCups (Cosmedent Inc., Chicago, Illinois, United States) to enhance surface gloss and integration, and the final restoration demonstrated excellent marginal adaptation, high luster, and seamless integration with surrounding enamel (Figure 6d).

Immediately after finishing and polishing, the sulcus-seated 2-mm-high modified Tofflemire band produced circumferential blanching that confirmed a stable sulcular seal throughout etching, bonding, and injection-molding of the preheated Filtek Supreme Ultra composite. Clinical inspection showed a flush cervical margin without gaps, overhangs, or marginal discoloration, and the emergence profile faithfully reproduced the original contour. Surrounding gingivae were pink, firm, and non-bleeding on probing, with no evidence of soft-tissue trauma or recession. The entire procedure, including local anesthesia, matrix customization, adhesive protocol, composite placement, and final polish, was completed uneventfully in a single appointment, obviating the need for rubber dam isolation or surgical crown lengthening. The patient reported no postoperative pain or sensitivity, expressed immediate satisfaction with the color match and anatomy, and required no occlusal or contour adjustments before dismissal. No intraoperative complications such as matrix displacement, contamination, or composite voids were observed. At the two-week follow-up, the restoration remained intact with healthy gingival margins and no bleeding or inflammation, and the patient reported continued absence of sensitivity or discomfort.

This case demonstrates a clinically successful restoration of a deep cervical lesion achieved through a conservative technique. By utilizing the modified matrix band for margin elevation and isolation, we obtained a restoration with proper contour and contact (though this lesion did not directly involve a broad proximal contact, the matrix ensured no composite overflow interproximally) and a healthy tissue response. The case demonstrates that even in an anterior tooth, a subgingival defect can be managed without resorting to periodontal surgery, provided that meticulous adhesive technique and isolation are employed.

## Discussion

This case highlights several important considerations in managing subgingival cervical lesions. Achieving a dry field and a well-adapted matrix is paramount for adhesive success in deep lesions [1,2]. Traditional matrices or wedges often fail when the cavity margin lies below the gumline, leading to excess composite and poor contour. In our case, a customized Tofflemire matrix effectively replaced the role of a surgical approach by physically retracting the gingiva and sealing the margin. This technique draws on principles from Brackett et al. [3], who used a modified matrix to isolate root caries, and from Chan and Adkins' approach to restoring subgingival cervical lesions and Mennito and Renne's simplified technique for interproximal root surface lesions [4,5]. By trimming the band to approximately 2 mm height and carefully inserting it (with slight gingival blanching as a positive sign), we created a temporary "wall" that contained our restorative materials. Notably, no rubber dam was used during the margin elevation phase; a rubber dam can be placed after elevating the margin, but in this case, the matrix itself sufficed for isolation.

Deep margin elevation provides a tissue-preserving alternative to surgical crown lengthening by relocating a subgingival margin coronally with adhesive procedures [6]. The broader proximal box elevation concept further supports using composite to move margins to a cleansable, finishable position when surgery would compromise aesthetics or add morbidity [7]. The choice of a resin composite in this case was guided by aesthetic demands, given that the lesion was on a visible canine, and the desire for strong adhesion to enamel.

A notable technique detail was preheating the composite resin. Warming the composite to approximately 68°C increases its flow without altering its chemistry, leading to improved marginal adaptation and potentially reduced microleakage [8]. Clinicians should be aware that once removed from the warmer, the composite begins cooling (and increasing in viscosity) within minutes, so it's important to work efficiently.

Our matrix modification also drew on the two-matrix maneuver for margin elevation when additional support is needed [9]. Follow-up data and reviews indicate that, when carefully executed, elevated composite margins can perform comparably to margins originally placed supragingivally [10]. The enamel present at the margins allowed for reliable bonding, and by using an etch-and-rinse adhesive protocol (total etch with chlorhexidine and a hydrophobic bonding resin), we aimed to maximize the seal, principles emphasized in classic guidance on adhesive cementation and margin quality [11].

RMGI is often recommended for subgingival or root surface lesions due to its fluoride release and tolerance to moisture. "Open-sandwich" strategies, where an RMGI base is placed beneath the composite for deep cervical restorations, have demonstrated acceptable longevity [12]. Both approaches are valid; a recent 36-month clinical trial comparing cervical lesions restored with microhybrid composite versus RMGI found no significant difference in success or retention, with both materials performing well [13]. That said, an RMGI-like base effect was achieved here by a thin (≈0.5 mm) flowable composite initial layer, which functioned as a stress-relieving/adaptation layer while preserving a fully adhesive composite restoration.

We observed that the warmed composite was easier to inject and packed nicely into the narrow subgingival space, ensuring the critical cervical margin was well sealed [8]. A common concern with deep margin elevation or any subgingival restoration is its impact on periodontal health. Violating the biologic width (the supracrestal attachment tissues) can trigger chronic inflammation and attachment loss. In this case, although the restoration margin remained about 1 mm below the gingival margin, it was still above the alveolar bone crest by an estimated 3 mm, meaning the biologic width was respected. Moreover, the finish of the restoration was smooth and flush, minimizing plaque retention. Reassuringly, evidence suggests that properly executed adhesive restorations at or slightly below the gumline do not necessarily harm periodontal health; teeth with adhesive fragment reattachments at subgingival fracture lines maintained healthy periodontal indices over two years [14]. In our patient, we similarly noted healthy gingiva at follow-up, with no increase in probing depth or bleeding, in line with the notion that a well-finished composite margin is biocompatible. Patients should be instructed in oral hygiene around the margins; using superfloss or floss threaders can help clean just below the contacts to prevent plaque accumulation.

Practical tips that aided our outcome included the following: using a slightly thicker or curved band (e.g., Greater Curve) to resist collapse; considering a "matrix-in-matrix" support if depth demands (not required here); performing a sequential cure, that is, cure the gingival composite increment through the band first and then remove the band and cure again directly on the composite, because metal bands attenuate light and the direct cure ensures complete polymerization; and placing a wedge after the first increment to push the composite tightly into the cervical area and compensate for slight matrix spring-back when the band is removed. Lastly, postoperative maintenance is important. The patient was placed on a fluoride rinse regimen to help prevent new root caries and was advised to avoid excessively hard brushing at the cervical area to prevent abrasion of the restoration. The longevity of such restorations depends on both the material's bond and the patient's home care, especially since root surfaces lack the self-remineralizing capacity of enamel.

## Conclusions

This case demonstrates that a modified Tofflemire matrix technique can successfully facilitate the restoration of a deep subgingival cervical lesion on an anterior tooth without surgical intervention. The key finding is that a trimmed and contoured matrix band can achieve deep sulcular isolation, allowing the proper adaptation of restorative materials in areas otherwise prone to moisture and bleeding contamination. This approach effectively relocates the cervical margin coronally, simplifying the subsequent restoration process. Utilizing an etch-and-rinse adhesive protocol and warmed composite resin, it is possible to obtain a well-sealed, anatomically correct restoration at the original subgingival margin. The immediate outcomes in this case showed excellent marginal integrity and periodontal health, indicating that the technique is biologically compatible when carefully executed. The modified matrix margin elevation technique serves as a minimally invasive alternative to surgical crown lengthening for subgingival lesions. It preserves tooth structure and soft tissues, avoids the drawbacks of surgery (such as bone removal and aesthetic changes), and can be completed in a single visit. Current evidence and this clinical example suggest that such adhesive restorations can be durable and safe for the periodontium over time.

Dentists should consider this technique when managing similar cases of cervical or proximal lesions below the gumline, especially in patients who prefer to avoid surgery or in aesthetically sensitive areas. Adhering to meticulous technique, including proper matrix modification, moisture control, and incremental curing, is critical to success. Further clinical reports and long-term studies will continue to inform best practices, but this case adds to the growing support for deep margin elevation strategies in restorative dentistry.
